# Tanshinone I, a new EZH2 inhibitor restricts normal and malignant hematopoiesis through upregulation of *MMP9* and *ABCG2*

**DOI:** 10.7150/thno.53170

**Published:** 2021-05-08

**Authors:** Ying Huang, Shan-He Yu, Wen-Xuan Zhen, Tao Cheng, Dan Wang, Jie-Bo Lin, Yu-Han Wu, Yi-Fan Wang, Yi Chen, Li-Ping Shu, Yi Wang, Xiao-Jian Sun, Yi Zhou, Fan Yang, Chih-Hung Hsu, Peng-Fei Xu

**Affiliations:** 1 Women's Hospital, and Institute of Genetics, Zhejiang University School of Medicine, Hangzhou, Zhejiang, China.; 2 State Key Laboratory for Medical Genomics, National Research Center for Translational Medicine at Shanghai, Shanghai Institute of Hematology, Rui-Jin Hospital affiliated to Shanghai Jiao-Tong University School of Medicine, Shanghai, China.; 3 Department of biophysics and Kidney Disease Center of the First Affiliated Hospital, School of Medicine, Zhejiang University, Hangzhou, Zhejiang, China.; 4 Women's Hospital, and Institute of Genetics, and Department of Environmental Medicine, Zhejiang University School of Medicine, Hangzhou, China.; 5 College of Pharmaceutical Sciences, Zhejiang University.; 6 Zhejiang University-University of Edinburgh united Institute.; 7 Key Laboratory of Adult Stem Cell Translational Research, Chinese Academy of Medical Sciences, National & Guizhou Joint Engineering Laboratory for Cell Engineering and Biomedicine Technique, Guizhou Province Key Laboratory of Regenerative Medicine, Guizhou Medical University, Guiyang, Guizhou, China, 550004.; 8 Harvard Department of Stem Cell and Regenerative Biology, Harvard University, Cambridge, MA 02138, USA.; 9 Stem Cell Program and Division of Hematology/Oncology, Boston Children's Hospital, Pediatric Hematology/Oncology at Dana Farber Cancer Institute, Harvard Stem Cell Institute, Harvard Medical School and Howard Hughes Medical Institute, Boston, MA 02115, USA.

**Keywords:** Tanshinone I, hematopoiesis, leukemia, EZH2, H3K27me3

## Abstract

**Rationale:** Tanshinone, a type of diterpenes derived from *salvia miltiorrhiza*, is a particularly promising herbal medicine compound for the treatment of cancers including acute myeloid leukemia (AML). However, the therapeutic function and the underlying mechanism of Tanshinone in AML are not clear, and the toxic effect of Tanshinone limits its clinical application.

**Methods:** Our work utilizes human leukemia cell lines, zebrafish transgenics and xenograft models to study the cellular and molecular mechanisms of how Tanshinone affects normal and abnormal hematopoiesis. WISH, Sudan Black and O-Dianisidine Staining were used to determine the expression of hematopoietic genes on zebrafish embryos. RNA-seq analysis showed that differential expression genes and enrichment gene signature with Tan I treatment. The surface plasmon resonance (SPR) method was used with a BIAcore T200 (GE Healthcare) to measure the binding affinities of Tan I. *In vitro* methyltransferase assay was performed to verify Tan I inhibits the histone enzymatic activity of the PRC2 complex. ChIP-qPCR assay was used to determine the H3K27me3 level of EZH2 target genes.

**Results:** We found that Tanshinone I (Tan I), one of the Tanshinones, can inhibit the proliferation of human leukemia cells *in vitro* and in the xenograft zebrafish model, as well as the normal and malignant definitive hematopoiesis in zebrafish. Mechanistic studies illustrate that Tan I regulates normal and malignant hematopoiesis through direct binding to EZH2, a well-known histone H3K27 methyltransferase, and inhibiting PRC2 enzymatic activity. Furthermore, we identified *MMP9* and *ABCG2* as two possible downstream genes of Tan I's effects on EZH2.

**Conclusions:** Together, this study confirmed that Tan I is a novel EZH2 inhibitor and suggested *MMP9* and *ABCG2* as two potential therapeutic targets for myeloid malignant diseases.

## Introduction

Epigenetic regulations play essential roles in both physiological and pathological processes. The specificity, reversibility, and dynamics of these controls make them ideal drug targets 1. One of the epigenetic regulators, histone lysine methyltransferase Enhancer of Zeste Homologue 2 (EZH2), is an enzymatic component of Polycomb repressive complex 2 (PRC2). It mediates transcriptional silencing through histone H3 lysine 27 methylation 2. EZH2 has been shown to play a pivotal role in both physiological functions and the pathogenesis of many cancers, including several types of leukemia 3. In the mouse model, the deletion of *Ezh2* leads to insufficient expansion of Hematopoietic Stem Cells (HSCs) and defective erythropoiesis in the fetal liver. The *Ezh2*-deficient mouse embryos died because of anemia 4. Methyltransferase EZH2 and demethylase JMJD3 antagonistically modulate HSCs activation by regulation *Bambi*, *Cdkn1a* and *Gadd45* 5. Overexpression of EZH2 results in the myeloproliferative disorder in mice, and has been shown to be associated with aggressiveness and metastasis of different types of human cancers such as prostate cancer, breast cancer, bladder cancer and melanoma 6-10. A recent study also identified the context-specific roles of EZH2 in Acute Myeloid Leukemia (AML) 11, 12, which is a rapidly progressive and poor-prognosis malignancy arising from hematopoietic stem cells. In this case, the therapeutic potential of targeting EZH2 has received considerable attention in leukemia/cancer management.

In recent years, natural products gained much attention in cancer therapy, many herbal medicinal products have been proved to have the function of anti-cancer 13. For example, Vibsanine A induces myeloid leukemia cell differentiation by activating PKC signaling 14. Hyperforin promotes apoptosis of cancer cells from various solid tumors and hematological malignancies, including B-cell chronic lymphocytic leukemia 15. Ingredients and compounds from natural herbs are huge drug resources, and the growing studies of their pharmacological mechanism will provide a better research foundation for the development of therapies.

Dan-shen (*Salvia miltiorrhiza Bunge*) is one of the most popular herbal remedies in China and several other Asian countries. The extracts of Dan-shen have been widely used for clinical treatment of coronary heart diseases, cerebral ischemia and early cirrhosis 16. Tanshinones are the major lipophilic ingredients of Dan-shen and have been shown to have diverse pharmacological activities including anti-inflammation, anti-oxidation and anti-cancer 17, 18. Among the main Tanshinones, Cryptotanshinone (CPT) inhibits the proliferation of mouse hepatoma cells and activates adaptive antitumor immune defenses 19. Tanshinone IIA could induce G1/G0 arrest, cell differentiation and apoptosis in human leukemia cell lines 20. Although Tanshinones' anti-cancer potentials have been more and more recognized, the molecular mechanism has not been well elucidated; mainly the molecular target of Tanshinone is still not well addressed.

In this study, we first looked at the toxicity and induced phenotype of five main ingredients of Dan-Shen in the zebrafish model. We then focused on one with the least toxicity but have significant effects on hematopoiesis, Tanshinone I (Tan I). Both human leukemia cell lines and zebrafish myeloid malignancies and xenograft models were utilized to study the pharmacological function and mechanisms of Tan I. We found that Tan I can inhibit both normal and malignant hematopoiesis by binding directly to EZH2 and subsequent repression of the histone H3K27 methyltransferase activity of PRC2 complex. Furthermore, we noticed that two known EZH2 downstream genes, *MMP9* and *ABCG2* are both up-regulated by Tan I treatment. The overexpression of these two genes in the human leukemia cell line can induce apoptosis and differentiation, suggesting the potential of these two genes for serving as novel targets for anti-leukemia therapies.

## Materials and methods

### Zebrafish lines and Reagents

Zebrafish are raised and maintained at 28.5 °C and on a 14 h light/10 h dark cycle at zebrafish core facilities of Zhejiang University School of Medicine. Transgenic lines* Tg(fli1:EGFP)* 21, *Tg(lyz:EGFP)* 22, *Tg(c-myb^hyper^:GFP*) 23,* Tg(lyz:EGFP);pu.1^G242D^* 24, and *Tg(hsp70l:ezh2:CG)* 25 were used in this study.

Tan I (T5330, Sigma-Aldrich), Tan IIA (T2906, Target Mol), Cpt (T2814, Target Mol), mmp9 inhibitor Mmp9-I (444278, Sigma-Aldrich), and ABCG2 inhibitor ko143 (K2144, Sigma-Aldrich) were dissolved in DMSO.

### WISH, Sudan Black and O-Dianisidine Staining

All WISH (Whole-mount *In situ* Hybridization) staining was performed with antisense digoxigenin-labeled RNA probes as previously described 26. Sudan Black and O-Dianisidine staining was performed as described previously 27, 28.

### Cell culture and Lentivirus transduction

HL-60 and NB4 cell lines were obtained from Cell Bank of Shanghai Institute of Hematology and cultured in RPMI-1640 medium (Invitrogen Corporation) supplemented with 10% FBS (Gibco-BRL) in 5% CO_2_ and humidified atmosphere at 37 °C.

Lentiviruses expressing MMP9-GFP and control GFP, ABCG2-mCherry and control mCherry were obtained from Genechem (Shanghai, China), and the infection of lentivirus on the NB4 cells was performed as described previously 29. Leukemia cells were counted and spin-infected with lentiviral particles, then cells were washed with PBS 12 h after infection. GFP^+^ or mCherry^+^ cells were isolated by FACS after three days and sorted again after five days.

### Flow cytometry analysis and cell sorting

All antibodies were purchased from BD PharMingen: PE-conjugated CD11b and APC-conjugated Annexin V. Total cells were Fc-blocked and stained with indicated combinations of antibodies for 30 min on ice, then washed three times and resuspended in 1% FBS/PBS. For apoptosis analysis, cells were resuspended with binding buffer and stained with Annexin V and 7-AAD for 15 min at 25 °C in the dark. The flow cytometric data were collected on a BD Calibur flow cytometer and analyzed using FlowJo software or Summit software. For the fluorescence-activated cell sorting (FACS), GFP^+^ or mCherry^+^ cells were sorted out by a cell sorter BD FACSAriaTM (BD Biosciences).

### Zebrafish xenograft model

Human NB4 or HL-60 cells were transplanted into 30 hpf (hours post-fertilization) zebrafish embryos as reported previously 30 with minor modifications: 3×10^6^ cells/mL of HL-60 or NB4 cells were collected and stained with DiI, then 200-300 cells were injected into 30 hpf dechorionated zebrafish embryos. After injection, embryos were incubated in 37 °C incubator and screened for positive fluorescence cells in the caudal hematopoietic tissue (CHT) after 24 h of the transplantation. Fluorescence cells were counted and analyzed using Image J.

### RT-qPCR and RNA-seq

Total RNAs were extracted using Trizol. RNA was reverse transcribed using Prime Script™ RT reagent Kit with gDNA Eraser (Perfect Real Time) Kit (RR047A, Takara). SYBR^®^ Premix Ex Taq™ II (Tli RNase H Plus) (RR820A, Takara) was used for the real-time qPCR analysis, with a CFX-Touch detection system (Bio-rad). *β-actin* was used as an internal control. Primer sequences are available in Supplementary Table S1.

72 hpf *Tg(c-myb^hyper^:GFP*) zebrafish embryos and NB4 cells treated with DMSO or Tan I were collected for RNA sequencing. Library construction and sequencing were completed by Novogene (Novogene Bioinformatics Technology Co., Ltd, Beijing, China). Paired-end sequencing (Novaseq6000, 150-bp reads) was performed. The sequencing reads were aligned to the zebrafish GRCz11 genome and human GRCh38 genome separately using STAR (https://github.com/alexdobin/STAR), and reads mapped to multiple genomic locations were removed. Gene expression counts for each sample were calculated by feature Counts 31. Differential expression analysis was performed by DESeq2 package (https://github.com/mikelove/DESeq2). Zebrafish and human homolog genes were identified using NCBI HomoloGene database (https://www.ncbi.nlm.nih.gov/homologene/). Transcription expression heatmap was performed by gplots (https://github.com/talgalili/gplots). GSEA (gene set enrichment analysis) was performed with GSEA software (http://software.broadinstitute.org/gsea/downloads.jsp). All the RNA-seq raw data have been deposited in Gene Expression Omnibus (GEO) database under the accession numbers GSE155572 and GSE155573.

### Morpholinos injection and heat shock

Morpholino Oligonucleotides (MOs) were purchased from Gene-Tools (https://www.gene-tools.com/). The sequences of MOs used in this study are following: *mmp9* translation-blocking MO: 5'-CGCCAGGACTCCAAGTCTCATTTTG-3'; *abcg2d* translation-blocking MO: 5'-AAGACAGCATTGTCAGGCATCTTCT-3'. All MOs were injected into embryos at one-cell stage.

*Tg(hsp70l:ezh2:CG)* dechorionated embryos at 30 hpf stage were placed in 15 mL Falcon centrifuge tubes and incubated at 37 °C or 28.5 °C (control group) water bath for 1 h, then the embryos were transferred back to dishes and incubate at 28.5 °C in the incubator.

### Western blotting

The protein of zebrafish embryos (72 hpf; *Tg(c-myb^hyper^:GFP*) treated with DMSO and Tan I were extracted in RIPA (P0013B, Beyotime Biotechnology) containing 0.3% SDS and protease inhibitor cocktails (04693116001, Roche). Histone H3 was used as protein-loading controls.

### Affinity measurement

The Surface Plasmon Resonance (SPR) method was used with a BIAcore T200 (GE Healthcare) to measure the binding affinities. Zebrafish EZH2(Detai Bio) or SUZ12(Detai Bio) was diluted in sodium acetate solution (pH 5.0) with a final concentration of 50 μg/mL. EZH2 or SUZ12 was immobilized on a CM5 sensor chip (GE Healthcare) by amine coupling to reach target densities of 12000 resonance units (RU). Immobilized EZH2 or SUZ12 was used to capture the chemical compound. Human EED (ProSpec-Tany) was diluted in sodium acetate solution (pH 5.0) with a final concentration of 200 μg/mL. EED was immobilized on a CM5 sensor chip (GE Healthcare) by amine coupling to reach target densities of 9100 resonance units (RU). The running buffer contained PBS-T (10 mM sodium phosphate, 150 mM NaCl, 0.005% Tween-20, pH 7.4) and 2% DMSO. Data were recorded at 25 °C.

Then injected 7 concentrations of the Tan I (0.78 µM, 1.56 µM, 3.125 µM, 6.25 µM, 12.5 µM, 25 µM, 50 µM) at a flow rate of 30 μL/min, the contact time was 2 min for EZH2 and SUZ12 and 2.5 min for EED, respectively. The dissociation time was 2 min for EZH2 and SUZ12 and 5 min for EED, respectively. EPZ6438 (0.78 µM, 1.56 µM, 3.125 µM, 6.25 µM, 12.5 µM, 25 µM) was monitored as positive control. A blank immobilization was performed for one of the sensor chip surfaces to use for correction of the binding response. Sensorgrams were analyzed using the Igor Pro version 6.1 (WaveMatrix).

### *In vitro* methyltransferase assay

A 0.28 μg wild type PRC2 complex (31387, Active Motif, Japan) was mixed with different concentrations of Tan I (2% DMSO as the control) in HMT buffer (50 mM Tris-HCl (pH 8.8), 0.02% Triton X-100, 2 mM MgCl_2_, 1 mM dithiothreitol), followed by pre-incubation at 30 °C for 30 min. The reaction mixtures were then added 0.2 μg recombinant histones H3.3 (M2507S, NEB, USA) or Hela core histones (53501, Active Motif, Japan) and 2 μL of 3.2 mM SAM (S-adenosyl-Lmethionine SAM B9003S, NEB, USA), followed by methylation reactions at 30 °C for 60 min. The final reaction mixtures were separated by 15% sodium dodecyl sulfate (SDS)-PAGE, and subjected to western blot analysis.

### Chromatin immunoprecipitation (ChIP)

ChIP assays for NB4 cells and zebrafish embryos were performed essentially as previously described 29, 32 with minor modifications. Briefly, cells were harvested and crosslinked with 1% formaldehyde for 10 min at room temperature. After sonication, the soluble chromatins were incubated with the following antibodies separately: anti-H3K27me3 (ab6002, Abcam); anti-EZH2 (ab3748, Abcam) or control IgG (ab172730, Abcam). Chromatin immunocomplexes were then precipitated with protein G (Millipore, 16-662). The immunoprecipitated complex was washed, and DNA was extracted and purified by QIAquick PCR Purification Kit (Qiagen). EZH2 ChIP-seq data for human LNCaP cells were obtained from GEO series GSE107780. ChIP-seq data were processed by Cistrome analysis pipeline and loaded to University of California-Santa Cruz genome browsers for visualization 33. ChIP DNA was analyzed by qPCR using specific primers, and the data were normalized by input DNA. The results were derived from three independent experiments. The primers used for ChIP-qPCR are listed in Supplementary Table S1.

### Statistics analysis

Data are expressed as mean ± SEM. Differences between the control and each treatment of antagonists were assessed by *t*-test analysis of variance using GraphPad Prism version 7.0 software. Differences with *p* < 0.05 were considered statistically significant.

## Results

### Tan I can inhibit normal and malignant definitive hematopoiesis in zebrafish embryos

The major ingredients of *Salvia miltiorrhiza* include lipid-soluble Tanshinones such as Tanshinone I (Tan I), Tanshinone IIA (Tan IIA) and Cryptotanshinone (Cpt), et.al, and water-soluble phenolic acids such as Salvianolic acid B, Danshensu and Rosmrinic acid, et.al 18. To examine the toxicity and treatment phenotypes using the zebrafish system, we tested the survival rate, morphology, vasculature and blood cells with the treatments of different ingredients at various concentrations in wild type and transgenic zebrafish (**Figure S1**). We found that water-soluble phenolic acids Salvianolic acid B and Danshensu have no obvious effect on treated zebrafish embryos. Instead, all lipid-soluble Tanshinones induced various degrees of developmental defects in the zebrafish embryo. Compared to Tan IIA and Cpt, Tan I treatment had the lowest fatality rate even at high concentrations (**Figure S1A**). For example, 30 μM Tan I treatment did not affect the embryonic vasculature **(Figure S1B)** but leads to an obvious decrease of the granulocytes in the 72 hpf embryos, showing by both Sudan Black B (SB) staining and *lyz* EGFP^+^ cells in *Tg(lyz:EGFP)* transgenic zebrafish (**Figure S1C-D**). We also evaluated the effect of Tanshinones on primitive hematopoiesis by whole mount *in situ* hybridization (WISH) of *scl*, *gata1* 34 and *hbαe1* 35 at either 20 hpf or 30 hpf following the treatment of the wild type zebrafish embryos with Tan I, Tan IIA and Cpt starting at 12 hpf (**Figure S2**). However, we didn't observe any obvious difference between the Tanshinones treated and DMSO treated embryos, suggesting a potential role of Tan I in the definitive hematopoiesis but not primitive hematopoiesis. In the following studies, we focused on the effects of Tan I on normal and malignant definitive hematopoiesis and explored its mechanisms.

**Figure 1:**
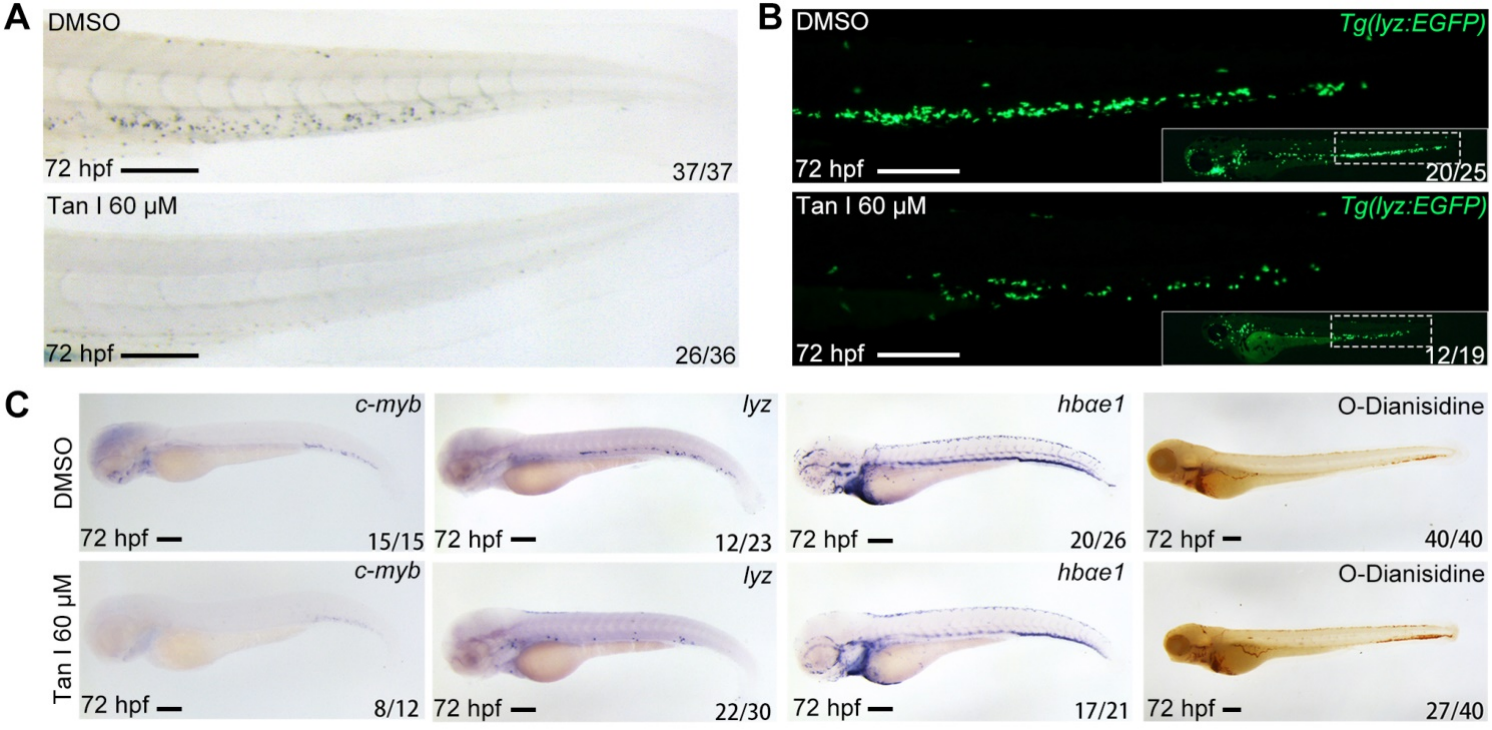
** Tan I treatment leads to a decrease of definitive hematopoiesis in zebrafish.** (**A**) Sudan Black staining of Caudal hematopoietic tissue (CHT) regions of the DMSO or Tan I treated embryos at 72 hpf. (**B**) Fluorescent images of CHT regions (the framed region of whole embryos in the right bottom) of the Tg*(lyz:EGFP)* zebrafish embryos at 72 hpf treated with DMSO or Tan I. (**C**) Images of the embryos treated with DMSO or Tan I WISH analyzed with *c-myb*, *lyz*, *hbαe1* probes or stained with O-Dianisidine. Scale bars: 200 µm.

To confirm the effect of Tan I on zebrafish definitive hematopoiesis, we treated the wild type and *Tg(lyz:EGFP)* transgenic zebrafish with 60 μM Tan I at 30 hpf and analyzed phenotypes at 72 hpf by Sudan Black staining, WISH and fluorescent microscopy. We found that Tan I can dramatically reduce the definitive hematopoietic stem/progenitor cells (HSPCs) number shown by WISH of *c-myb* 36 (**Figure 1C**) as well as the myelopoiesis shown by SB staining (**Figure 1A**), *lyz* EGFP^+^ cells (**Figure 1B**) and WISH of *lyz* 37 (**Figure 1C**)*.* But there was only some minor impact on the erythrocytes shown by WISH of *hbae1* and O-Dianisidin staining (**Figure 1C**).

**Figure 2:**
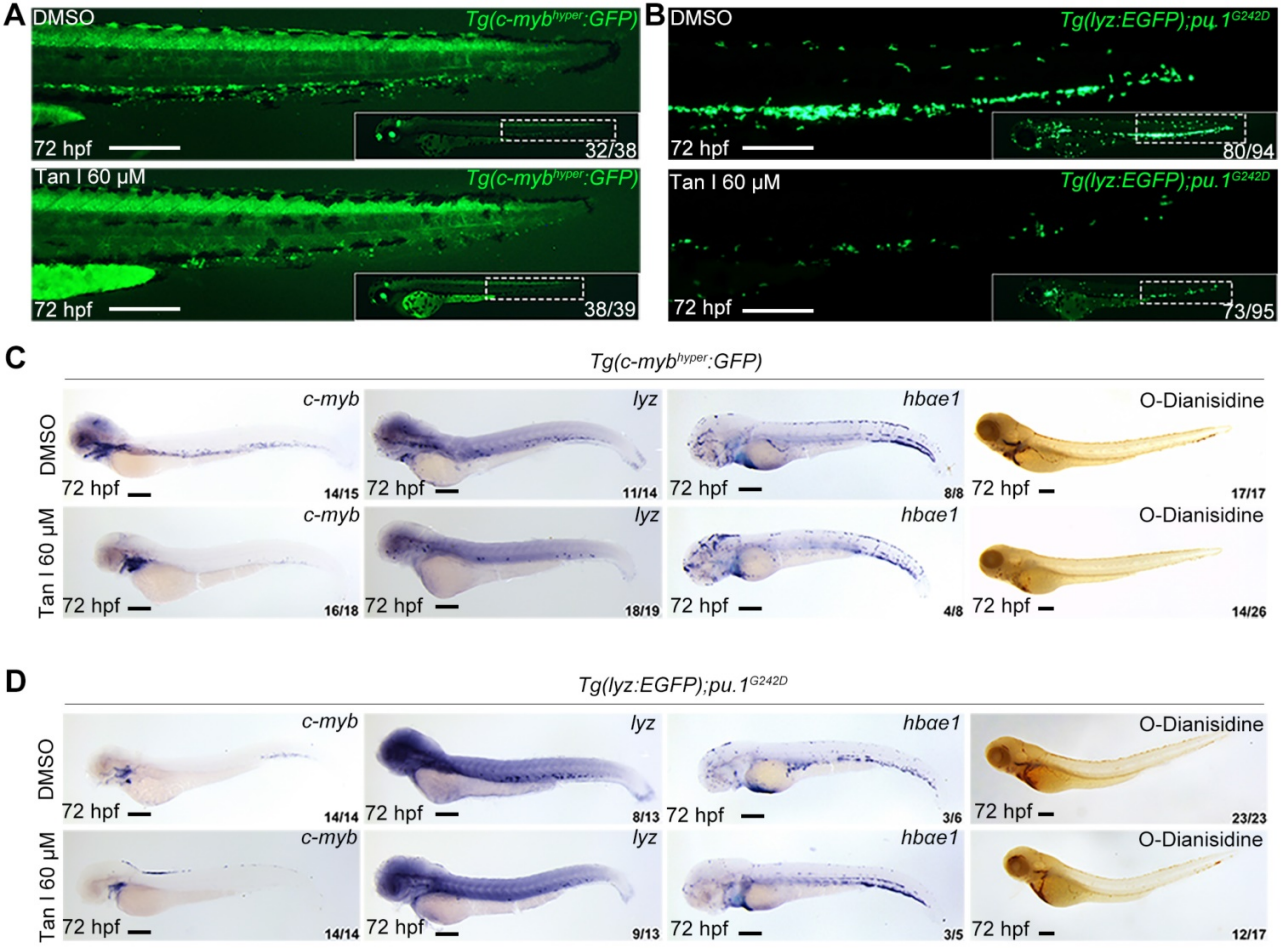
** Tan I treatment can inhibit the expanded myelopoiesis in *c-myb* hyperactive and *pu.1*^G242D^ zebrafish.** (**A**) Fluorescent images of *Tg(c-myb^hyper^:GFP)* zebrafish embryos with DMSO or Tan I treatments. (**B**) Fluorescent images of *Tg(lyz:EGFP);pu.1^G242D^* zebrafish embryos treated with DMSO or Tan I. (**C**) Images of *Tg(c-myb^hyper^:GFP)* embryos treated with DMSO or Tan I by -*c-myb*, *lyz*, and *hbαe1* WISH and O-dianisidine staining. (**D**) Images of *Tg(lyz:EGFP);pu.1^G242D^* embryos treated with DMSO or Tan I evaluated by *c-myb*, *lyz*, and *hbαe1* WISH or O-dianisidine staining. All treatments were continuously starting from 30 hpf. Embryos in all images were at 72 hpf of development. Scale bars: 200 µm.

To further test if the inhibitory effect of Tan I exhibited in malignant hematopoiesis, we used a high concentration of Tan I to treat two previously reported zebrafish leukemia models, c-*MYB* hyperactive (*c-myb*^hyper^) zebrafish 23 and *pu.1* hypomorphic (*pu.1*^G242D^) zebrafish 24. Both models were shown to have abnormal expansion of myelopoiesis and respond to chemotherapy agents. We found that the embryos were morphologically normal after Tan I treatment, but compared to the DMSO group, the Tan I treatment led to a dramatic decrease of the *c-myb* GFP^+^ cells in *c-myb*^hyper^ fish (**Figure 2A**) and *lyz* EGFP^+^ cells in *pu.1*^G242D^ zebrafish (**Figure 2B**), indicating an inhibitory effect of Tan I on malignant hematopoiesis. To confirm such effect at a molecular level, WISH of *c-myb*, *lyz*, *hbαe1* and O-Dianisidin staining were performed in treated embryos of the two transgenics. Consistent with the effects observed in wild type embryos, Tan I treatment could largely restrict the expression of *c-myb* and *lyz*. Again, it had only minor effects on erythrocytes, in both *c-myb*^hyper^ fish (**Figure 2C**) and *pu.1*^G242D^ fish (**Figure 2D**).

### Tan I inhibits human leukemia cell growth *in vitro* and in the xenograft zebrafish model

**Figure 3:**
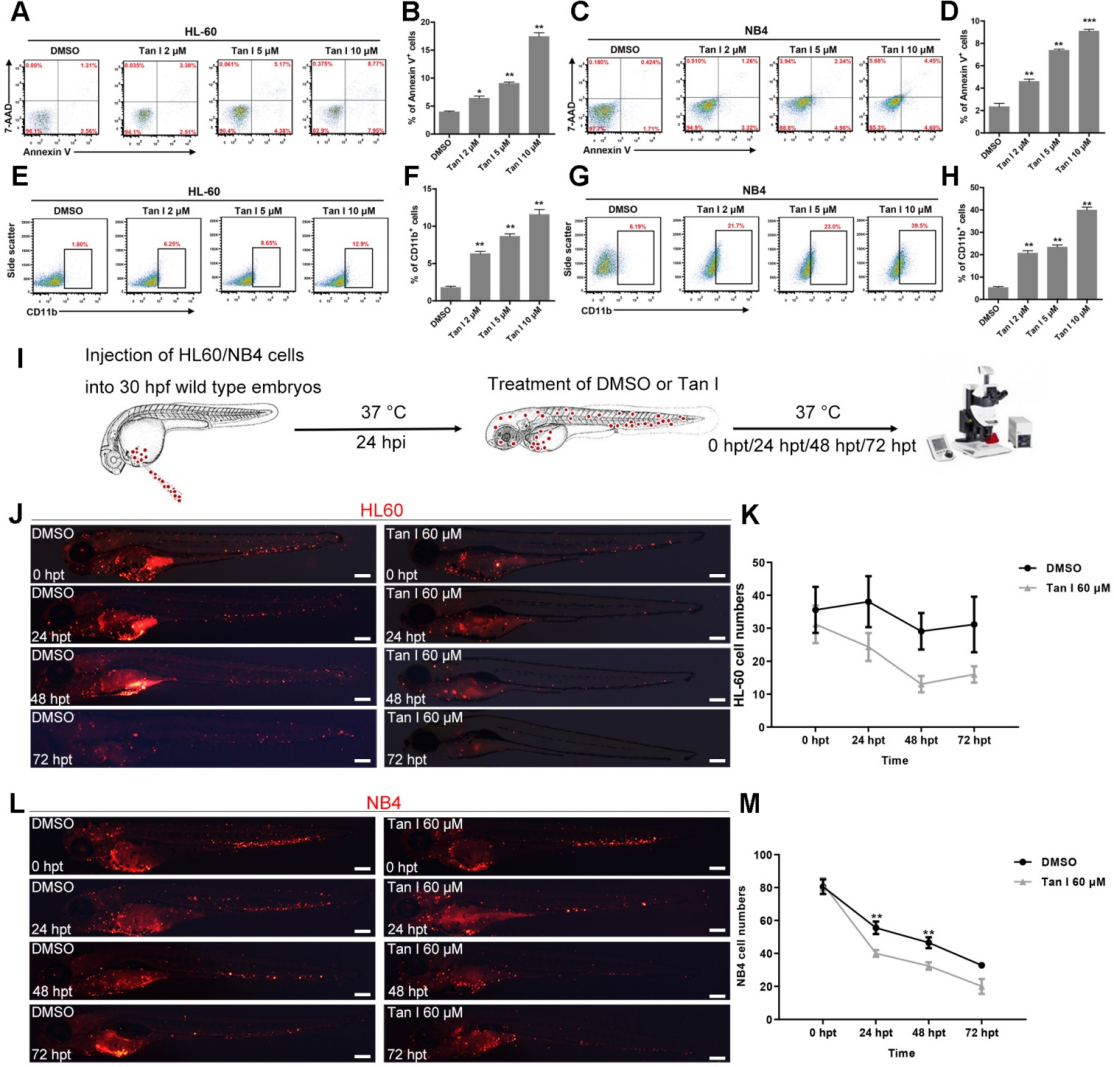
** Tan I inhibits the proliferation of human leukemia cell lines *in vitro* and in the zebrafish xenograft model.** (**A-D**) Flow cytometric analyses of Annexin V and 7-AAD staining after treatments of HL-60 (A, B) or NB4 (C, D) cells with DMSO or the indicated concentrations of Tan I for 3 days. (**E-H**) Flow cytometric analyses of CD11b expression after treatments of HL-60 (E, F) or NB4 (G, H) cells with DMSO or the indicated concentrations of Tan I for 3 days. (**I**) Cartoon illustrating steps of the embryonic zebrafish xenograft assay, hpi (hours post-injection), hpt (hours post-treatment). (**J**) Fluorescent images of HL-60-cell grafted zebrafish embryos at indicated times post transplantation with DMSO or Tan I treatments. (**K**) Statistic analysis of HL-60 cell number in the CHT regions of images in panel J, Error bars represent standard error of means (SEM). (**L**) Fluorescent images of NB4-cell grafted zebrafish embryos at indicated times post transplantation with DMSO or Tan I treatments. (**M**) Statistic analysis of NB4 cell number in the CHT regions of embryos shown in panel L, Error bars represent standard error of means (SEM). Scale bars: 200 µm.\

The above *in vivo* results in zebrafish models suggested that Tan I could be a potential drug for treating leukemia. Therefore, we further tested this hypothesis in two human leukemia cell lines: HL-60 (M2, C-Myc amplification) cells 38 and NB4 (M3, PML-RARa^+^) cells 20. As measured by the percentages of the apoptotic Annexin V^+^ cells and the differentiated CD11b^+^ myeloid cells, Tan I treatment significantly induced leukemic apoptosis and myeloid differentiation in both cell lines, which also exhibited a dose-dependent effect according to the concentration of Tan I (**Figure 3A-H**).

Xenograft models are one of the gold standards for assessing the pre-clinical efficacy of cancer drugs. In recent years, due to its rapid growth, easy of imaging and the ability to perform large numbers of transplantations, zebrafish xenograft approaches where human cancer cells were transplanted into embryos at larval stages gained lots of attention 39. To further test the potential of Tan I as a drug for treating leukemia, we transplanted HL-60 cells and NB4 cells into the yolk of zebrafish embryos at 30 hpf, and treated the xenografted embryos with DMSO or Tan I at 24 h after the transplantation (**Figure 3I**). We found that both HL-60 and NB4 cells could enter circulation and home to the CHT region of zebrafish embryos (**Figure 3J, L**), although HL-60 cells survived well in the CHT, the NB4 cell number dropped more quickly during the embryonic development. Compared to DMSO, Tan I treatment significantly reduced the grafted cell number in the CHT region over time in both the zebrafish xenograft models (**Figure 3K-M**).

### Tan I binds directly to EZH2 and inhibits PCR2 enzymatic activity

**Figure 4:**
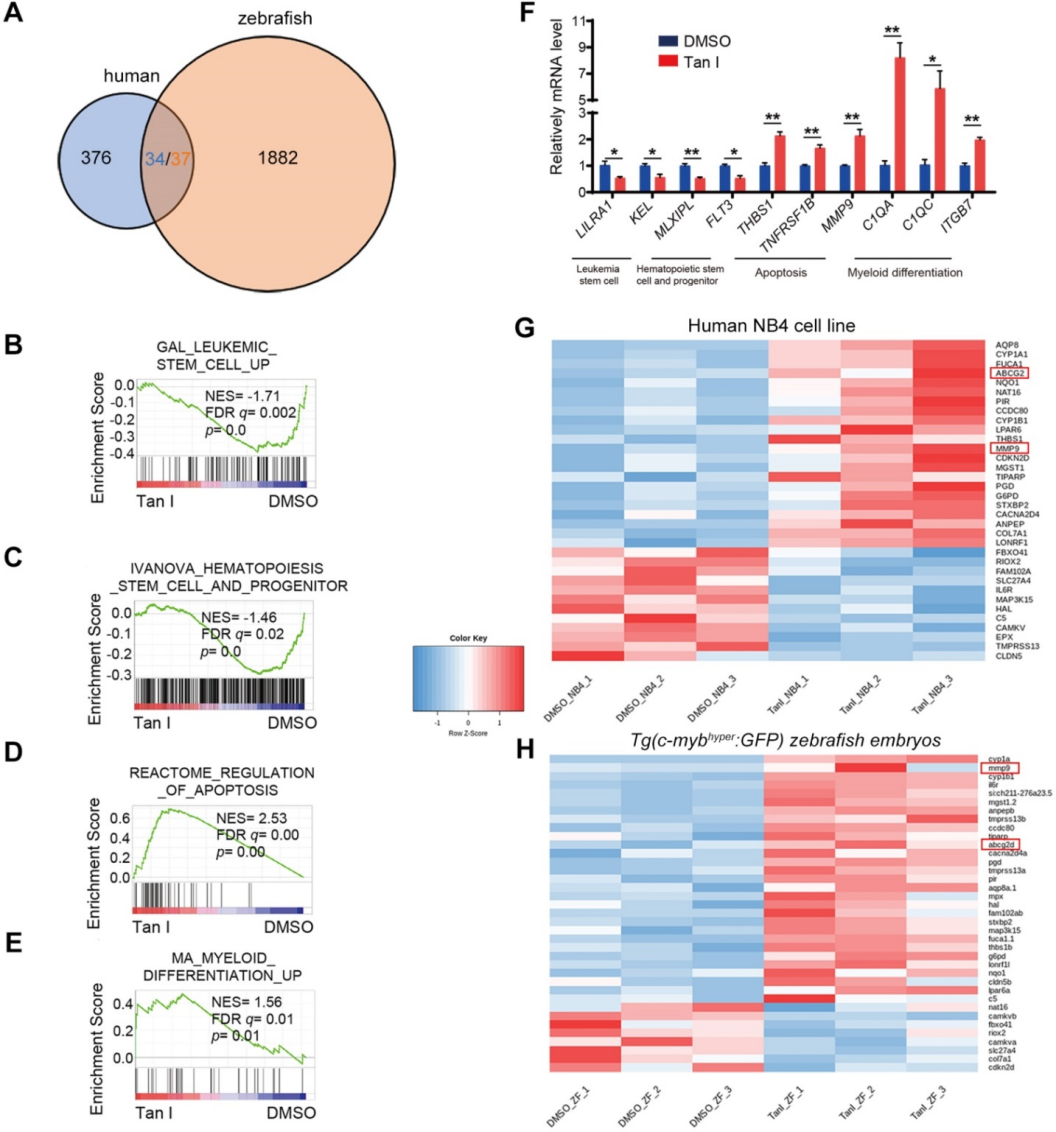
** Transcriptomic analysis of Tan I-treated human leukemia cell line and zebrafish.** (**A**) Venn diagram showing the numbers of differentially expressed genes between DMSO and Tan I treated NB4 cell (blue) or* Tg(c-myb^hyper^:GFP)* zebrafish (orange), the overlapped gene number also shown (three of the human genes have two homologs each in zebrafish). (**B-E**) GSEA (Gene Set Enrichment Analysis) of the expressing profile of NB4 cells treated with DMSO or Tan I using a leukemia stem cell-associated upregulated signature (B), a hematopoietic stem cell and progenitor-associated signature (C), an apoptosis-associated signature (D) and a myeloid differentiation-associated upregulated signature (E). (**F**) RT-qPCR assay on the mRNA levels of a number of genes after treatments of NB4 cells with DMSO or 10 µM Tan I for 3 days. (**G-H**) Heatmap of 36 differentially expressed genes between DMSO and Tan I treatment in both human NB4 cell (G) and *Tg(c-myb^hyper^:GFP)* zebrafish (H).

**Figure 5:**
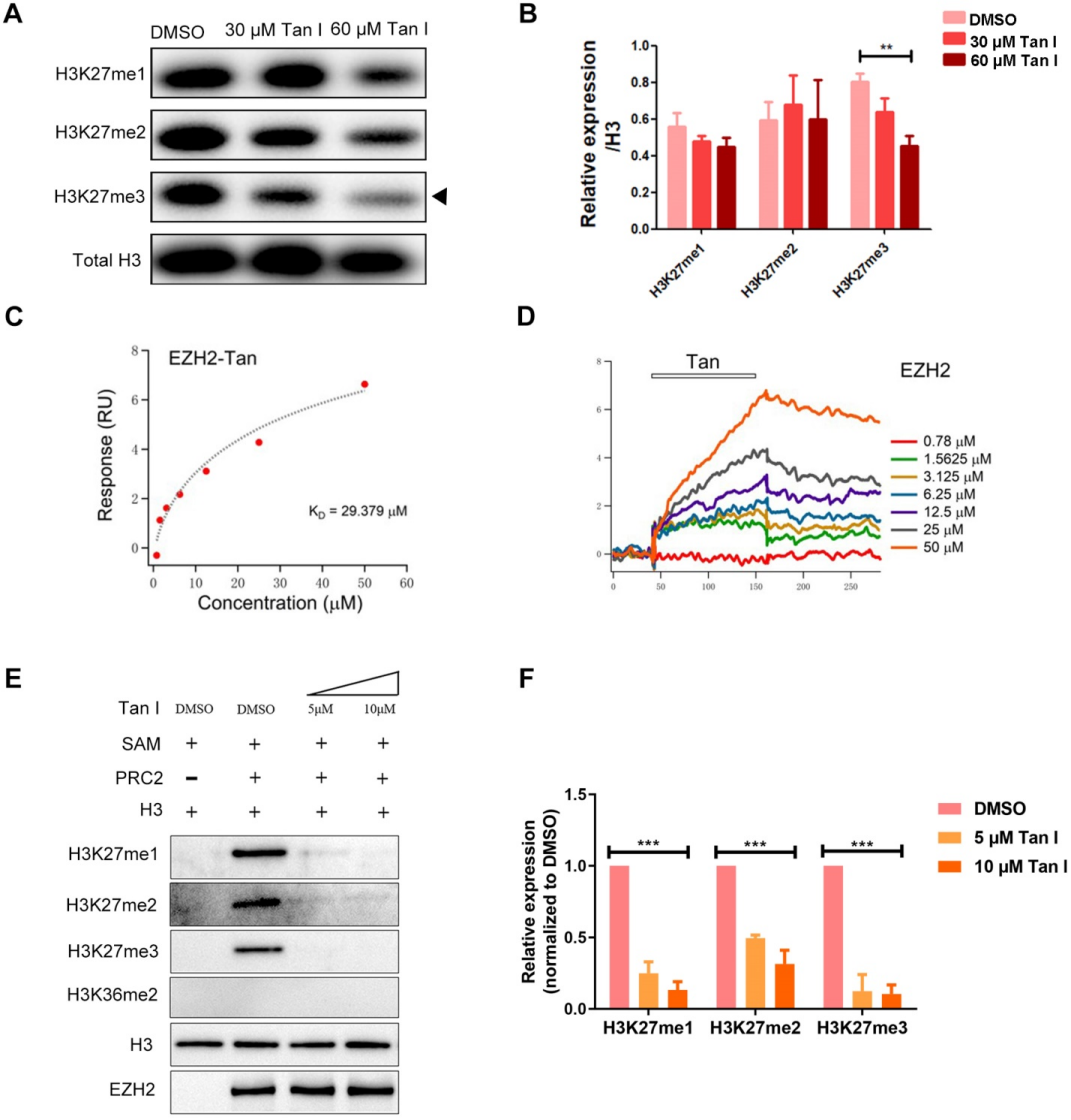
** Tan I binds to EZH2 directly and inhibit PCR2 enzyme activity.** (**A-B**) *c-myb^hyper^* zebrafish embryos were treated with DMSO or indicated concentrations of Tan I. The histone H3K27me1, me2, me3 levels were measured by western blotting, total histone H3 was used as the control. (B) is the quantification of the western blotting. (**C-D**) SPR analysis of the binding between EZH2 and Tan I at different concentrations. The open bar in (D) indicated the duration of Tan I perfusion in SPR. (**E-F**) *In vitro* inhibition of PRC2 complex activities by Tan I. In detail, the indicated proteins (0.28 μg of the PRC2 complex and 0.2 µg of histone H3) were mixed with Tan I and SAM. The reaction products were separated by 15% SDS-PAGE and analyzed by western blotting using antibodies against histone H3K27me1, me2, me3, H3K36me2, histone H3, and EZH2.

To explore the mechanism of how Tan I inhibits malignant hematopoiesis at the gene expression level, we performed RNA-sequencing (RNA-seq) analysis both in the NB4 cells and in the *c-myb*^hyper^ fish after the treatment of DMSO or Tan I. We found that 376 genes were differentially expressed between DMSO and Tan I treatment in the NB4 cells and 1882 differentially expressed genes in *c-myb*^hyper^ fish embryos (*p* value ≤0.05, |log_2_ fold change| ≥ 0.25) (**Figure 4A**). Consistent with the observation above, the gene set enrichment analysis (GSEA) revealed negative enrichment for the gene signature associated with leukemia stem cell as well as hematopoietic stem cell and progenitor, and positive enrichment for the gene signature associated with apoptosis and myeloid differentiation in Tan I-treated NB4 cells (**Figure 4B-E, Figure S3A-B**), and the representative gene expression was validated by qPCR assay (**Figure 4F**). In the differentially expressed genes of the NB4 cells and the *c-myb*^hyper^ fish embryos, there were 34/37 (three of the human genes have two homologs each in zebrafish) overlapped genes between human cells and zebrafish (**Figure 4A**). Among these genes, two of them caught our attention, *MMP9* and *ABCG2,* whose ectopic expression were reported to result in the defects in hematopoiesis 40-43, were both up-regulated by Tan I treatment (**Figure 4G-H, Figure S3C**). Interestingly, both of them were under the regulation of EZH2 through the histone H3K27 methylation 40,44. Therefore, we hypothesized that the inhibitory function of Tan I on hematopoiesis might work through epigenetic mechanisms, possibly by regulating histone methylation. To test this hypothesis, we measured the histone H3K4, H3K9, H3K36 and H3K27 methylation levels in zebrafish embryos after Tan I treatment by western blotting. We found that compared to DMSO, Tan I treatment led to a significant decrease of the H3K27me3, H3K27me2 also was slightly reduced by Tan I treatment, but there was no noticeable change in other histone methylations (**Figure S4 and Figure 5A-B**). Importantly, we also found that Tan I had no impact on EZH2 RNA and protein expression levels (**Figure S3C-D**).

Histone H3K27me3 is mainly regulated by the PRC2 complex in which EZH2 is the main catalytic subunit. To test whether the decrease of H3K27me3 level caused by Tan I was due to the direct inhibition on EZH2, we carried out surface plasmon resonance (SPR) assay and found that the Tan I could directly bind to EZH2 protein fixed on the surface of the sensor chip and this binding follows a concentration-dependent manner (**Figure 5C-D**). We determined the K_D_ of Tan I binding to be 29.379 μM, which was consistent with the concentration range we employed in the *in vitro* and* in vivo* experiments. Compared to EPZ6438 (a reported EZH2 inhibitor), Tan I showed more strongly binding to EZH2 (**Figure S5A**). Meanwhile, we also tested whether Tan I can bind to the EED or SUZ12 protein (another component of PRC2 complex). Interestingly, SPR assay showed that Tan I binds with a much higher affinity (29 μM) (**Figure 5C**) to zEZH2 than to human EED (hEED) and hardly binds to zSUZ12 proteins, because the K_D_ of Tan I binding to hEED is 94.475 μM (**Figure S5B**), while zSUZ12a and zSUZ12b are 1518 μM and 762 μM (**Figure S5C-E**). These results suggested that Tan I could directly bind to PRC2 complex, but have higher affinity to EZH2 protein.

To further verify whether this binding resulted in inhibition of the histone enzymatic activity of the PRC2 complex, *in vitro* methyltransferase assay was performed. The recombinant PRC2 complex displayed the substrate specificity for histone H3K27 but not H3K36. Independent of the EZH2 protein level, 5 μM Tan I was sufficient to block PRC2-mediated methylation on H3K27 using H3.3 *in vitro* (**Figure 5E-F**). Similar results were presented that 10 μM Tan I could strongly block PRC2-mediated methylation on H3K27me3 using HeLa core histones *in vitro* (**Figure S5F**). Together, these results illustrated that Tan I was a bona fide inhibitor of the PRC2 complex by directly binding to EZH2, which is consistent with the results from previous report 45.

### Tan I inhibits malignant hematopoiesis through the inhibition of EZH2 activity and up regulation of EZH2 downstream genes *MMP9* and *ABCG2*

**Figure 6:**
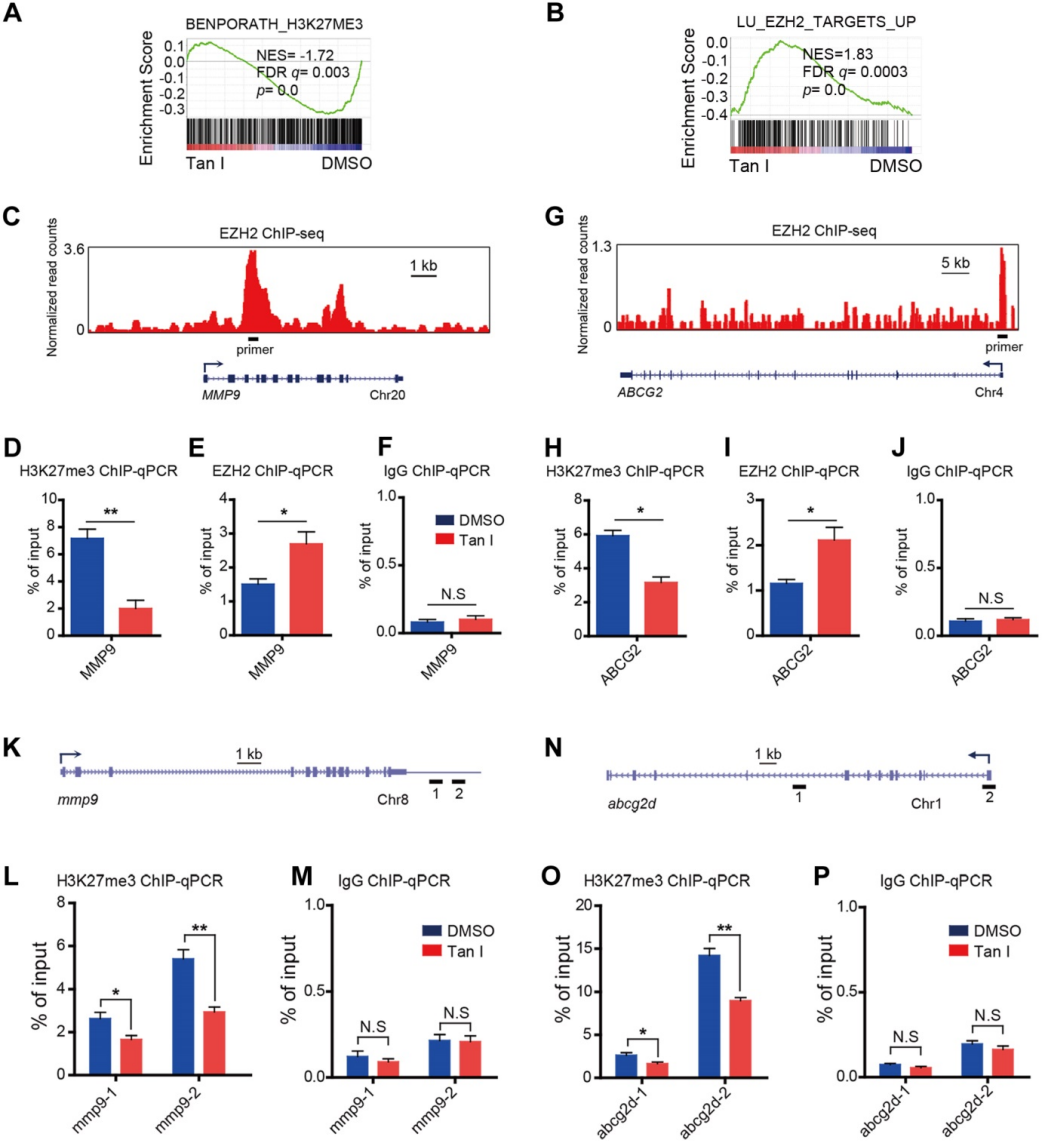
** Tan I affects the H3K27me3 modifications at the regulatory regions of the EZH2 downstream genes. (A-B)** GSEA of the expression profile of NB4 cells treated with DMSO or Tan I using a histone H3K27me3-associated signature “BENPORATH_H3K27ME3” (A) and an EZH2 target genes-associated signature “LU_EZH2_TARGETS_UP (M2139)” (B). (**C**) Genome browser track representing the binding sites of EZH2 at *MMP9* gene locus in human LNCaP cells. (**D-F**) ChIP-qPCR assay of H3K27me3 (D), EZH2 (E) or IgG (F) occupancy at *MMP9* gene locus in NB4 cells treated with DMSO or 10 µM Tan I for 3 days. (**G**) Genome browser track representing the binding sites of EZH2 at *ABCG2* gene locus in human LNCaP cells. (**H-J**) ChIP-qPCR assay of H3K27me3 (H), EZH2 (I) or IgG (J) occupancy at the *ABCG2* gene locus in NB4 cells treated with DMSO or 10 µM Tan I for 3 days. (**K**) Horizontal lines with Arabic numeral 1 to 2 indicate the regions at the *mmp9* gene locus amplified by qPCR in zebrafish ChIP assay. (**L-M**) ChIP-qPCR assay for H3K27me3 (L) or IgG (M) occupancy at the *mmp9* gene locus in zebrafish embryos treated with DMSO or 60 µM Tan I for 3 days. (**N**) Horizontal lines with Arabic numeral 1 to 2 indicate the regions at the *abcg2d* gene locus amplified by qPCR in zebrafish ChIP assay. (**O-P**) ChIP-qPCR assay for H3K27me3 (O) or IgG (P) occupancy at the *abcg2d* gene locus in zebrafish embryos treated with DMSO or 60 µM Tan I for 3 days. Data are presented as the mean ± SEM. *p < 0.05, **p < 0.01. All results are from three independent experiments.

EZH2 is a histone H3K27-specific methyltransferase. Tan I could directly bind to EZH2 and inhibit EZH2-mediated methylation of H3K27, we expected that the H3K27me3 level at the regulatory regions of direct target genes would decrease after Tan I treatment. As expected, GSEA revealed a negative enrichment for the gene signatures associated with the H3K27me3 in Tan I-treated NB4 cells (**Figure 6A**). Besides, GSEA exhibited enrichment of EZH2-bound targets in Tan I-treated NB4 cells (**Figure 6B**). The above results suggested that the up-regulation of *MMP9* and *ABCG2* by Tan I treatment might be due to the decrease of the PRC2 mediated H3K27me3 methylation. To validate this hypothesis, we performed Chromatin Immunoprecipitation-qPCR (ChIP-qPCR) assay both in the NB4 cell line and in the *c-myb*^hyper^ zebrafish embryos. We found that Tan I treatment significantly reduced the H3K27me3 level at the regulatory regions of both *MMP9* and *ABCG2* in the NB4 cells. Interestingly, the EZH2 binding of these regions was increased upon Tan I treatment. This increase could be explained by a feedback compensation of the H3K27me3 reduction (**Figure 6C-J**). A similar result was also observed in zebrafish. Tan I treatment led to a significant decrease of H3K27me3 level in both the *mmp9* and *abcg2d* regulatory regions (**Figure 6K-P**).

**Figure 7:**
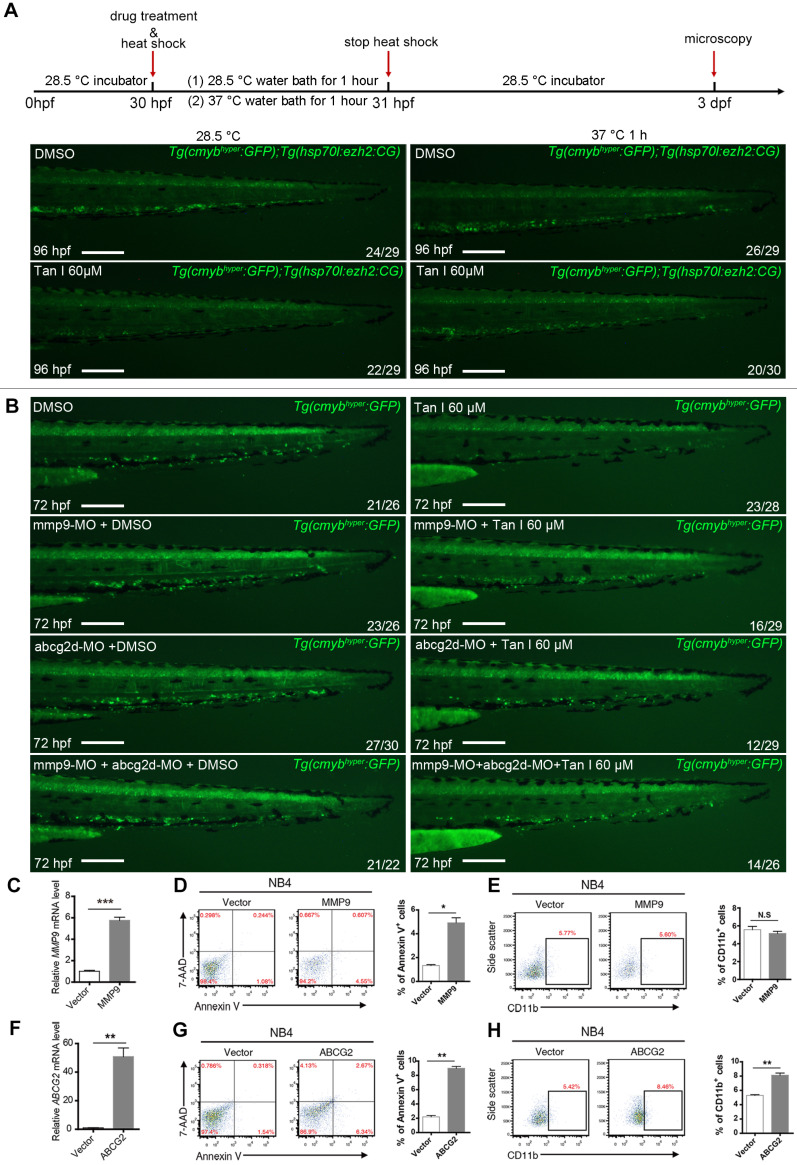
** Rescue experiment in zebrafish and overexpression experiment in the human leukemia cell line by *ezh2* or its downstream genes *mmp9* or *abcg2*.** (**A**) Rescue Tan I induced phenotype by overexpression of *ezh2* in the zebrafish embryo. Fluorescent images of *c-myb* GFP^+^ cells in *Tg(c-myb^hyper^:GFP) and Tg(hsp70l:ezh2:CG)* double transgenic zebrafish embryos, with DMSO or Tan I treatments and with or without a 37 ºC heat shock for 1h, as indicated. dpf (days post-fertilization) (**B**) Rescue Tan I induced phenotype by knockdown of *mmp9* and *abcg2* in the zebrafish embryo. Fluorescent images of *c-myb* GFP^+^ cells in *Tg(c-myb^hyper^:GFP)* transgenic zebrafish embryos. As indicated, *mmp9* and *abcg2d* morpholino was injected separately or together in DMSO or Tan I treated embryos. (**C-E**) Overexpression *MMP9* in NB4 cells. RT-qPCR analyses of *MMP9* mRNA level on the established NB4 cells transduced with an empty vector or *MMP9*-expressing vector (C). Flow cytometric analyses of Annexin V/7-AAD staining (D) and CD11b expression (E) in control or *MMP9*-overexpressed NB4 cells; (**F-H**) Overexpression of *ABCG2* in NB4 cells. RT-qPCR analyses of *ABCG2* mRNA level on the established NB4 cells transduced with empty vector or *ABCG2*-expressing vector (F). Flow cytometric analyses of Annexin V/7-AAD staining (G) and CD11b expression (H) in control or *ABCG2*-overexpressed NB4 cells. Scale bars: 200 µm.

To further investigate whether the hematopoietic inhibition of Tan I was through inhibition of EZH2 and its downstream genes *mmp9* and *abcg2*, we firstly used a previously reported heat-shock promotor driven *ezh2* overexpression transgenic zebrafish (*Tg(hsp70l:ezh2:CG)*) 25 to rescue the phenotype induced by Tan I. We found that after heat shock for 1 h at 37 ºC, the *c-myb* GFP^+^ cell number of most (2/3) Tan I-treated embryo can be recovered (**Figure 7A**). In addition, as the expression of *mmp9* and *abcg2d* are up-regulated by Tan I treatment, knockdown of those two genes may rescue the Tan I induced a decrease of *c-myb* GFP^+^ cells. To validate this hypothesis, we injected *mmp9* and *abcg2d* morpholino separately or together into the *c-myb*^hyper^ zebrafish embryos, and then treat them with 60 μM of Tan I. We found that knockdown of *mmp9* and *abcg2d* can partially rescue the *c-myb* GFP^+^ cell number (**Figure 7B**). We also used the inhibitors of these two genes to rescue the Tan I induced phenotype, although the inhibitors can somehow rescue the decreasing of *c-myb* GFP^+^ cell number induced by the treatment of Tan I at 30 μM (**Figure S6**), they were not adequate to rescue the Tan I induced phenotypes at 60 μM.

To investigate the function of *MMP9* and *ABCG2* in human leukemia cells, we overexpressed these two genes in the NB4 cells. The overexpression of both genes leads to increased apoptosis of the NB4 cells, interestingly, *ABCG2* overexpression can induce the myeloid differentiation of NB4 cells, but *MMP9* overexpression cannot (**Figure 7C-H**), suggesting that *ABCG2* may also be involved in other signaling pathway to restrict malignant myelopoiesis.

## Discussion

Although Tanshinones' anti-cancer activities have been shown before 18, the unclear mechanisms of the pharmacodynamics limit their clinical applications. Here we demonstrated that Tan I, one of the major Tanshinones, is a novel EZH2 inhibitor, and could repress both normal and malignant hematopoiesis *in vitro* and *in vivo*.

EZH2 is a key modulator of the balance between HSC self-renewal and differentiation. We found that normal hematopoiesis is severely affected in Tan I-treated zebrafish embryos, as evidenced by the reduction of the numbers of HSPCs and myeloid cells in zebrafish. Our results are consistent with a previous study that that Ezh2-deficient mice lead to insufficient expansion of HSPCs in fetal liver 4. In addition to its involvement in normal hematopoiesis, EZH2 as either an oncogene or tumor suppressor in various hematological malignancies 46, 47. It acts as a tumor suppressor in MDS and T-cell acute lymphoblastic leukemia (T-ALL), while also acts as an oncogene in AML and diffuse large B-cell lymphomas (DLBCL) 47. In DLBCL, EZH2 inhibition induces B-cell differentiation and maturation by promoting BLIMP1 expression 48, 49. Moreover, EZH2 inhibition reduces leukemia stem cells and enhances myeloid differentiation in AML 50. Consistently, we found that Tan I treatment restricts the transcriptional program of leukemia stem cell and promotes myeloid differentiation in AML cells. These observations implicate the existence of complex and diverse function of EZH2 on stem cell self-renewal, differentiation and proliferation, whose dysregulation contributes to the carcinogenesis in a cancer cell type-dependent manner.

We found that Tan I treatment could inhibit the definitive hematopoiesis of zebrafish but had no effect on primitive hematopoiesis, there was no obvious change of the overall morphology and vasculature either. These results indicated that, at least in the zebrafish embryo, the phenotypes induced by the Tan I treatment was specifically restricted in the hematopoietic system. Then we tested the therapeutic function of Tan I using published hematopoietic malignant zebrafish models, human leukemia cell lines and zebrafish xenograft models. All these studies showed that Tan I could effectively inhibit malignant hematopoiesis and leukemia cell growth. Mechanistic studies showed that Tan I treatment resulted in the up-regulation of *MMP9* and *ABCG2*, two genes that were reported to be associated with hematopoiesis and under the regulation of EZH2. Importantly, we found that Tan I could directly bind to EZH2 and inhibit PRC2 complex activity, and the global H3K27me3 of zebrafish dramatically reduces after Tan I treatment. In addition, the phenotypes induced by Tan I could be partially rescued by both up-regulation of EZH2 and down-regulation of *mmp9* and *abcg2* in zebrafish. We also demonstrated that overexpression of *MMP9* and *ABCG2* in the leukemia cell line could induce apoptosis and differentiation, indicating that these two genes could be potential targets for AML therapy.

Epigenetic regulations are dynamic and reversible processes that are critical in the establishment of normal cellular and developmental program, and aberrant epigenetic changes can lead to various human diseases like cancers 51. EZH2 is one of the most important epigenetic regulators which has been shown to be involved in multiple types of cancer. EZH2 inhibitors have drawn great attention to pharmaceutical research 3. Our work showed that targeting EZH2 can effectively suppress leukemia cell growth both *in vitro* and *in vivo*, but at the same time, we also notice that it has negative impacts on physiological functions such as normal hematopoiesis. This suggests that in the clinical application, the use of the EZH2 inhibitor should be carefully controlled and monitored, especially paying attention to its side effects on the hematopoietic system.

Tanshinone is one of the many ingredients from the valuable traditional Chinese herbs library, its therapeutic function has long been recognized. Our work utilized cell lines and animal models to elucidate the mechanisms of its pharmacological function. Our findings would provide information for better clinical application of Tanshinone I. However, there are remaining questions such as what is the binding site of Tan I to EZH2 protein, how the specificity of this binding is achieved, what are the differences and similarities between different Tanshinones as their chemical structural formula are similar, and is Tan I effective in other types of cancer cells? Further studies are still needed to address those questions.

## Supplementary Material

Supplementary figures and tables.
