# Supplementary material for: Tanshinone I, a new EZH2 inhibitor restricts normal and malignant hematopoiesis through upregulation of MMP9 and ABCG2
Source: Theranostics. 2021 May 8;11(14):6891–904. doi: 10.7150/thno.53170 (PMC8171091; doi:10.7150/thno.53170)
Supplement: Supplementary file 1 — Supplementary figures and tables. [file thnov11p6891s1.pdf]

Figure S1. Toxicity and phenotypes under treatments of different Tanshinones in zebrafish. (**A**) Survival rate analysis of zebrafish embryos treated by DMSO, Tan I, Tan IIA and Cpt at indicated concentrations. Treatments started at 12 hpf and statistics were done at day 3. (**B**) Fluorescent images of *Tg(fli1:EGFP)* transgenic zebrafish embryos treated with DMSO, Tan I, Tan IIA and Cpt at the indicated concentration. (**C**) Sudan Black staining of DMSO, Tan I, Tan IIA and Cpt at indicated concentration, the bottom is the statistical analysis. (**D**) Fluorescent images of *Tg(lyz:EGFP)* transgenic zebrafish embryos treated with DMSO, Tan I, Tan IIA and Cpt at the indicated concentration. All the treatments (B-D) started at 30hpf. Scale bars: 200 μm.

Figure S2. Treatments with Tanshinones have no effect on zebrafish primitive hematopoiesis. WISH of *scl, gata1, hbαe1* of embryos treated with DMSO, Tan I, Tan IIA and Cpt at the indicated concentration. All treatment started at 12 hpf and embryos were collected at the indicated stages. Scale bars: 200 μm

Figure S3. Validation of RNA-seq result. (A-B) GSEA of the expressing profile of NB4 cells treated with DMSO or Tan I using an apoptosis-associated signature (A) and a myeloid cell differentiation-associated signature (B). **(C)** Real-time PCR analysis of the RNA expression level of *abcg2d*, *ezh2*, *mmp9*, *nqo1*, *pgd and pir* in zebrafish embryos treated with DMSO or Tan I 60 μM. (**D**) The protein expression level of EZH2 in zebrafish embryos treated with DMSO or Tan I 60 μM

Figure S4. Histone methylation evaluation in Tan I treated zebrafish embryos. The indicated histone H3 methylation levels of DMSO- or Tan I-treated embryos were measured by western blotting, total histone H3 was used as control. Numbers on the top of each lane represent different biological repeats.

Figure S5. Tan I binds with a much higher affinity to zEZH2 than to hEED and hardly binds to zSUZ12 proteins and block PRC2-mediated methylation on H3K27me3 of HeLa core histones *in vitro*. (**A**) SPR analysis of the binding between EZH2 and EPZ6438 at different concentrations. (**B**) SPR analysis of the binding between hEED and Tan I at different concentrations. (**C**) SPR analysis of the binding between zSUZ12a and Tan I at different concentrations. (**D**) SPR analysis of the binding between zSUZ12b and Tan I at different concentrations. (**E**) Comparison of the binding response in SPR analysis of EZH2-Tan I, zSUZ12a-Tan I and zSUZ12b-Tan I at different concentrations. (**F**) *In vitro* inhibition of PRC2 complex activities by Tan I. In detail, the indicated proteins (0.28 μg of the PRC2 complex and 0.2 μg of HeLa core histones) were mixed with Tan I and SAM. The reaction products were separated by 15% SDS–PAGE and analyzed by western blotting using antibodies against histone H3K27me3, histone H4, and EZH2.

Figure S6. Rescue Tan I induced phenotype using Mmp9 and Abcg2 inhibitor. (**A-H**) Rescue Tan I induced phenotype by inhibiting Mmp9 and Abcg2d. Fluorescent images of *c-myb* GFP^{+} cells in *Tg(c-myb^{hyper}:GFP)* transgenic zebrafish embryos. As indicated, Mmp9 inhibitor mmp9-I (C-D) and Abcg2d inhibitor ko143 (E-F) were treated separately or together (G-H) in DMSO or Tan I treated embryos. Scale bars: 200 μm

Table S1. Sequences of PCR primers used in the study.

Figure S1

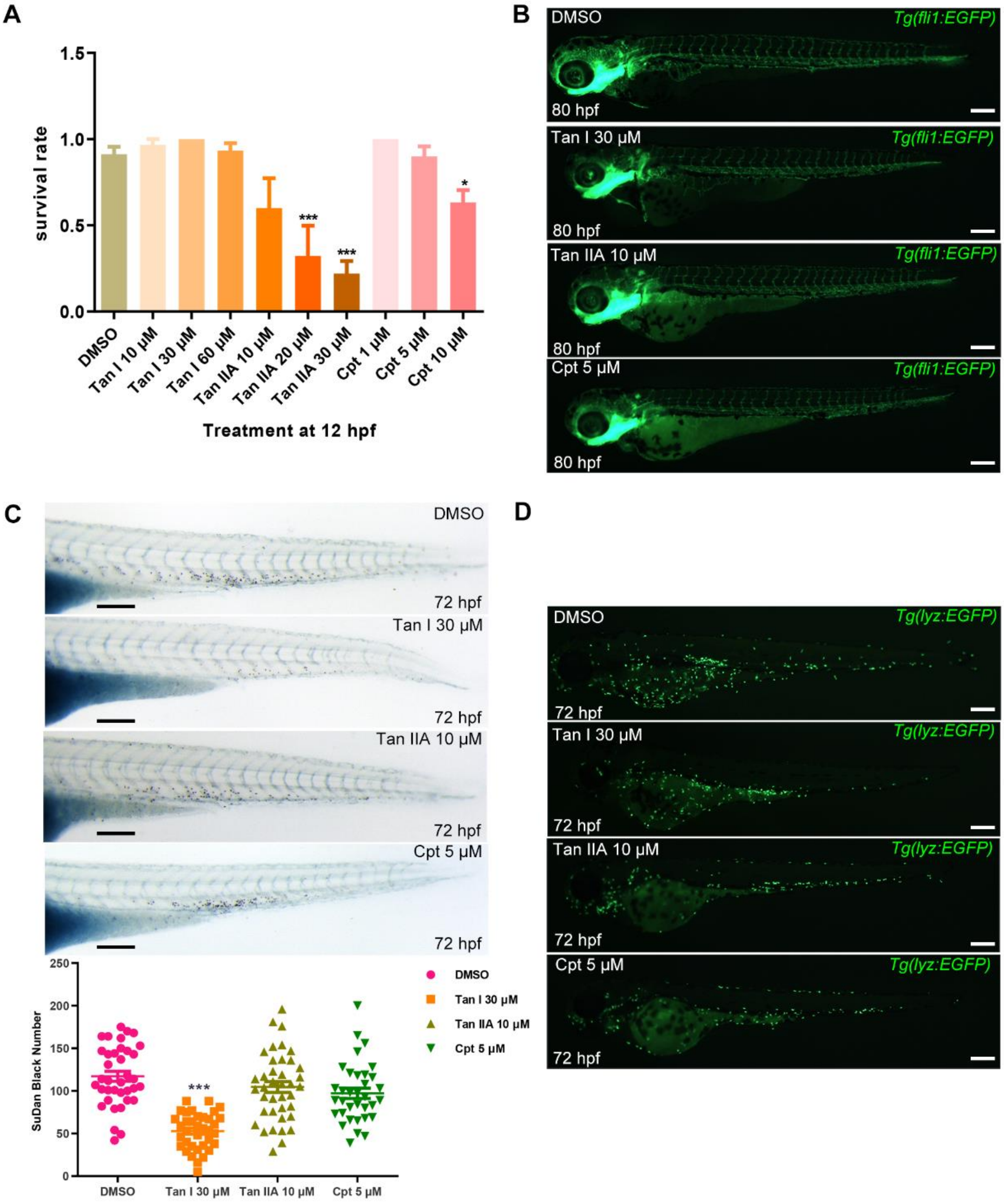
A
B
C
D
survival rate
1.5
1.0
0.5
0.0
***
***
*
DMSO
Tan I 10 μM
Tan I 30 μM
Tan I 60 μM
Tan IIA 10 μM
Tan IIA 20 μM
Tan IIA 30 μM
Cpt 1 μM
Cpt 5 μM
Cpt 10 μM
Treatment at 12 hpf
DMSO
Tg(fli1:EGFP)
80 hpf
Tan I 30 μM
Tan IIA 10 μM
Cpt 5 μM
72 hpf
Tg(lyz:EGFP)
SuDan Black Number
250
200
150
100
50
0
Tan I 30 μM
Tan IIA 10 μM
Cpt 5 μM

Figure S2

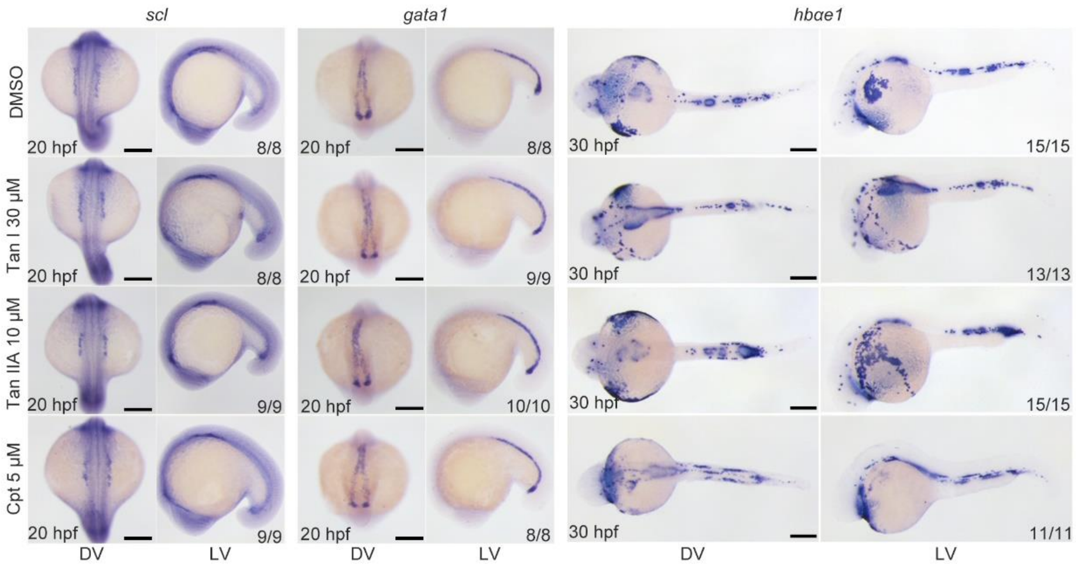

Figure S3

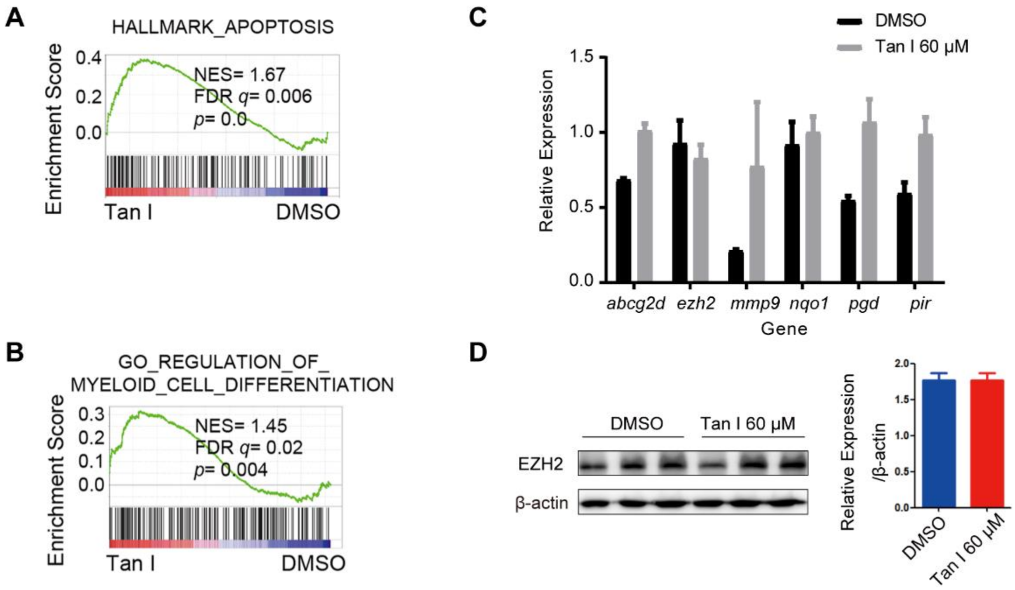

Figure S4

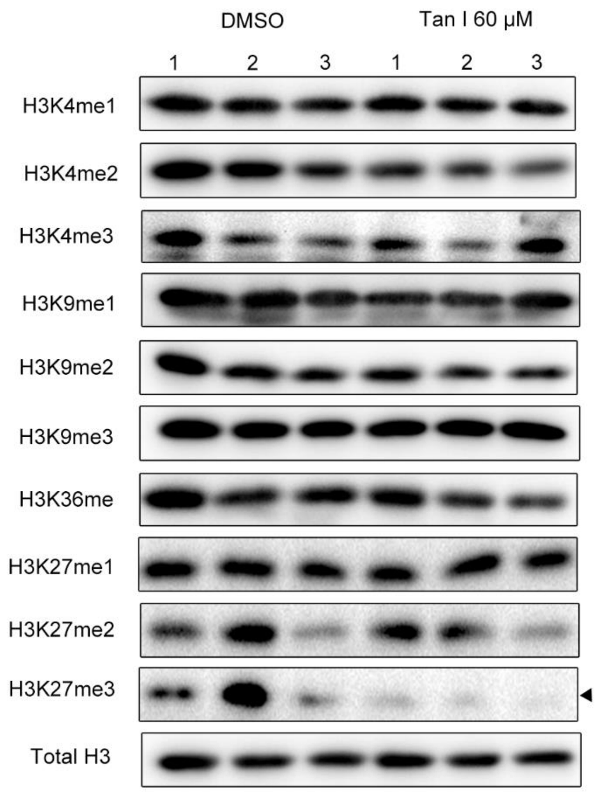

Figure S5

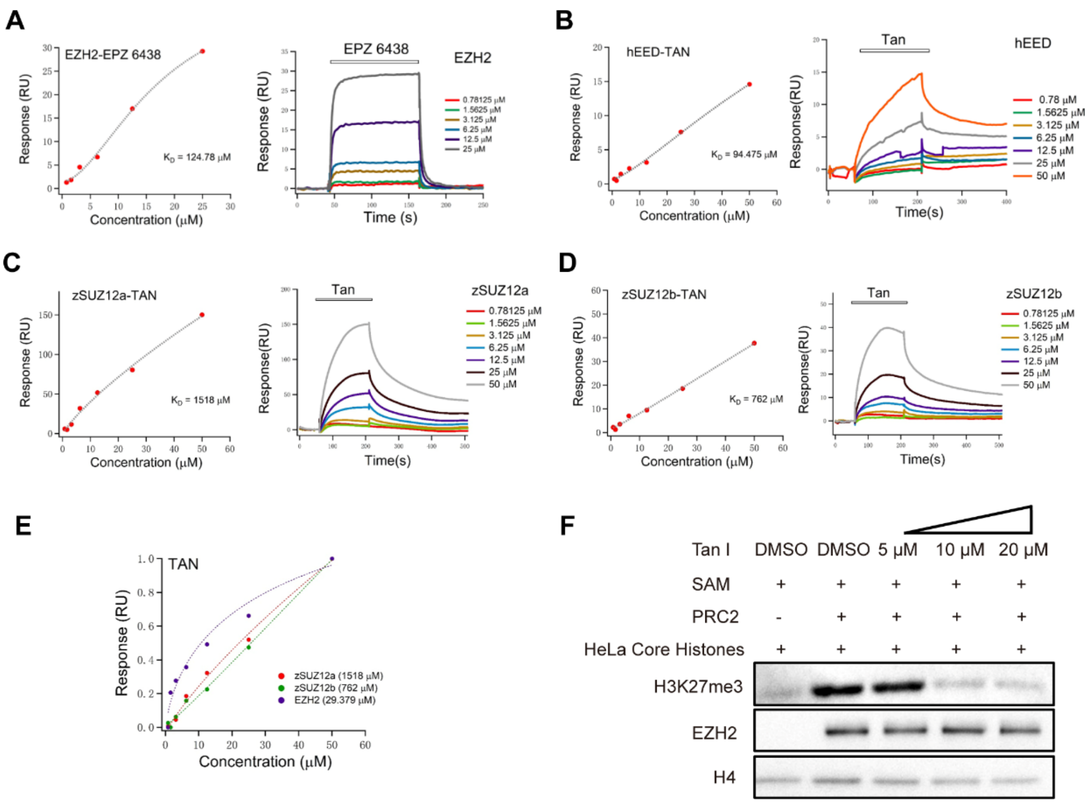

Figure S6

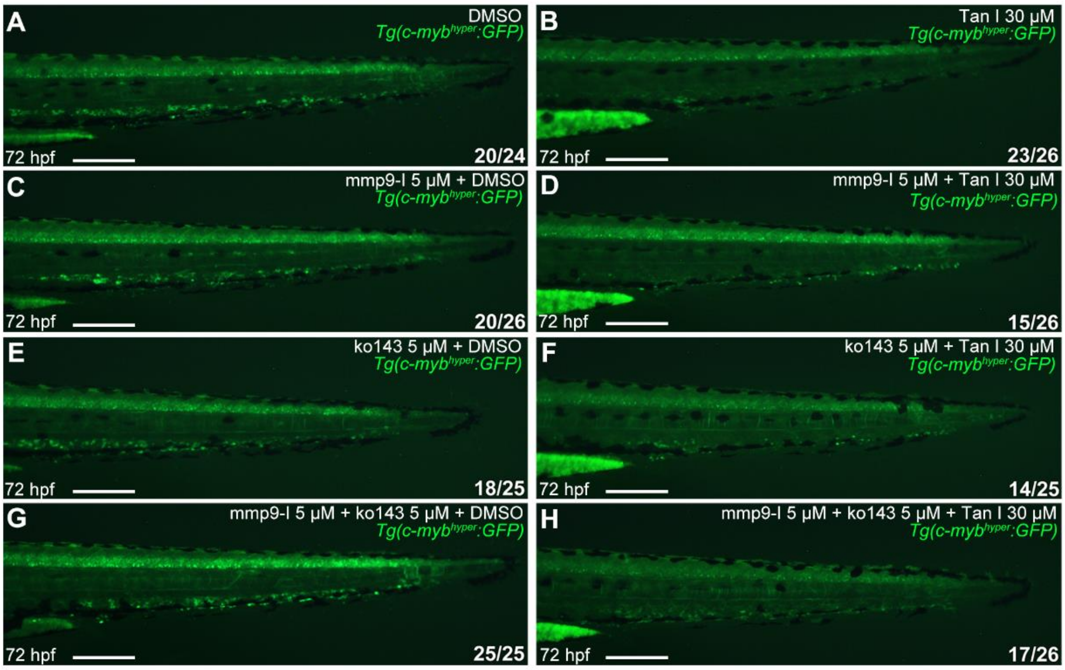
A
DMSO
Tg(c-myb^hyper:GFP)
72 hpf
20/24
B
Tan I 30 μM
Tg(c-myb^hyper:GFP)
72 hpf
23/26
C
mmp9-I 5 μM + DMSO
Tg(c-myb^hyper:GFP)
72 hpf
20/26
D
mmp9-I 5 μM + Tan I 30 μM
Tg(c-myb^hyper:GFP)
72 hpf
15/26
E
ko143 5 μM + DMSO
Tg(c-myb^hyper:GFP)
72 hpf
18/25
F
ko143 5 μM + Tan I 30 μM
Tg(c-myb^hyper:GFP)
72 hpf
14/25
G
mmp9-I 5 μM + ko143 5 μM + DMSO
Tg(c-myb^hyper:GFP)
72 hpf
25/25
H
mmp9-I 5 μM + ko143 5 μM + Tan I 30 μM
Tg(c-myb^hyper:GFP)
72 hpf
17/26

Table S1

Supplementary Table S1: Sequences of PCR primers used in the study.				
Gene	species	Test	Forward	Reverse
MMP9	Human	RT-qPCR	5’-TGTACCGCTATGGTTACACTCG-3’	5’-GGCAGGGACAGTTGCTTCT-3’
ABCG2	Human	RT-qPCR	5’-ACGAACGGATTAACAGGGTCA-3’	5’-CTCCAGACACACCACGGAT-3’
LILRA1	Human	RT-qPCR	5’-CCAGTGTGTTTCTGATGTCAGC-3’	5’-CCGGATGCACCGAGATGAAG-3’
KEL	Human	RT-qPCR	5’-CGGGTGCTGACAGCTATCC-3’	5’-ACACACAGATGTCTCACAGGG-3’
MLXIPL	Human	RT-qPCR	5’-AGAACCGGCGTATCACACAC-3’	5’-GTGCTCACGAGCCCATGAA-3’
FLT3	Human	RT-qPCR	5’-AGGGACAGTGTACGAAGCTG-3’	5’-GCTGTGCTTAAAGACCCAGAG-3’
THBS1	Human	RT-qPCR	5’-AGACTCCGCATCGCAAAGG-3’	5’-TCACCACGTTGTTGTCAAGGG-3’
TNFRSF1B	Human	RT-qPCR	5’-TTCATCCACGGATATTTGCAGG-3’	5’-GCTGGGGTAAGTGTACTGCC-3’
C1QA	Human	RT-qPCR	5’-TCTGCACTGTACCCGGCTA-3’	5’-CCCTGGTAAATGTGACCCTTTT-3’
C1QC	Human	RT-qPCR	5’-AGGATGGGTACGACGGACTG-3’	5’-GTAAGCCGGGTTCTCCCTTC-3’
ITGB7	Human	RT-qPCR	5’-AGAATGGCGGAATCCTCACCT-3’	5’-TGAAGTTCAGTTGCTTGCACC-3’
GAPDH	Human	RT-qPCR	5’-GGAGCGAGATCCCTCCAAAAT-3’	5’-GGCTGTTGTCATACTTCTCATGG-3’
MMP9	Human	ChIP-qPCR	5’-TTCAGCCCGCACTTATTTCG-3’	5’-CGAGAGGAAGAGGGTTAGCC-3’
ABCG2	Human	ChIP-qPCR	5’-CGCGTCTCTCAATCTCAGTG-3’	5’-GTGCTGTGCCCACTCAAAAG-3’
mmp9-1	Zebrafish	ChIP-qPCR	5’-ATTAATCACATCGCCATTCG-3’	5’-AGGGGTTTCCACGAGCTAAT-3’
mmp9-2	Zebrafish	ChIP-qPCR	5’-TTGTTGGTGTGCTCGACATT-3’	5’-AACACCTCTCCTCCTGCTGA-3’
abcg2d-1	Zebrafish	ChIP-qPCR	5’-CATGTGCTCGGCTTAACTCC-3’	5’-GGCTGTGCATCTTGAACCAA-3’
abcg2d-2	Zebrafish	ChIP-qPCR	5’-TGTTGTTCTCCTGCAAACGG-3’	5’-GGCAGAACGGGTCAAAGATC-3’
mmp9	Zebrafish	RT-qPCR	5'-ACAACCACTGCTTCCACCACAAC-3'	5'-TGCGTTCACCATTGCCTGAGATC-3'
abcg2d	Zebrafish	RT-qPCR	5'-GGAGAGAGGAAGAGGACCAGCATC-3'	5'-TGAGCAGCAGCAGCACAGAATG-3'
ezh2	Zebrafish	RT-qPCR	5'-GAGGCAAACTCTCGCTGTCAGAC-3'	5'-GCACCGCTCCAGTCCACATTC-3'
pgd	Zebrafish	RT-qPCR	5'-CAAGGACGCAGACGGCACTAAC-3'	5'-GACGAGAGGCATCTGGCAAAGAC-3'
pir	Zebrafish	RT-qPCR	5'-TCGCAGGAGTATTGGACGGAGAG-3'	5'-CACGGTGAGGATGATCAGGAAAGC-3'
nqo1	Zebrafish	RT-qPCR	5'-GAGAGGCTGGGTGGTGTGTTTG-3'	5'-TGATGTGCTGTGGTAATGCCGTAG-3'
β-actin	Zebrafish	RT-qPCR	5'-ATCAGGGTGTCATGGTTGGT-3'	5'-CACGCAGCTCGTTGTAGAAG-3'
